# Pronounced Postmating Response in the *Drosophila* Female Reproductive Tract Fluid Proteome

**DOI:** 10.1016/j.mcpro.2021.100156

**Published:** 2021-09-29

**Authors:** Caitlin E. McDonough-Goldstein, Emma Whittington, Erin L. McCullough, Sharleen M. Buel, Scott Erdman, Scott Pitnick, Steve Dorus

**Affiliations:** 1 Center for Reproductive Evolution, Department of Biology, Syracuse University, Syracuse, New York, USA; 2 Department of Biology, Syracuse University, Syracuse, New York, USA

**Keywords:** reproduction, fertility, secretion, fat body, mass spectrometry, translation, FDR, false discovery rate, FRT, female reproductive tract, GO, gene ontology, PC, principal component, PSMs, peptide–spectrum matches, SFP, seminal fluid protein, TCEP, tris(2-carboxyethyl)phosphine, YPD, yeast extract–peptone–dextrose

## Abstract

Fertility depends on the progression of complex and coordinated postmating processes within the extracellular environment of the female reproductive tract (FRT). Molecular interactions between ejaculate and FRT proteins regulate many of these processes, including sperm motility, migration, storage, and modification, along with concurrent changes in the female. Although extensive progress has been made in the proteomic characterization of the male-derived components of sperm and seminal fluid, investigations into the FRT have remained more limited. To achieve a comparable level of knowledge regarding female-derived proteins that comprise the reproductive environment, we utilized semiquantitative MS-based proteomics to study the composition of the FRT tissue and, separately, the luminal fluid, before and after mating in *Drosophila melanogaster*. Our approach leveraged whole-fly isotopic labeling to delineate female proteins from transferred male ejaculate proteins. Our results revealed several characteristics that distinguish the FRT fluid proteome from the FRT tissue proteome: (1) the fluid proteome is encoded by genes with higher overall levels of FRT gene expression and tissue specificity, including many genes with enriched expression in the fat body, (2) fluid-biased proteins are enriched for metabolic functions, and (3) the fluid exhibits pronounced postmating compositional changes. The dynamic mating-induced proteomic changes in the FRT fluid inform our understanding of secretory mechanisms of the FRT, serve as a foundation for establishing female contributions to the ejaculate–female interactions that regulate fertility, and highlight the importance of applying proteomic approaches to characterize the composition and dynamics of the FRT environment.

## Highlights

• *Drosophila* FRT tissue and luminal fluid proteomes were characterized.
• Whole-fly isotopic labeling was used to identify only female proteins.
• Postmating responses were significantly enriched within the fluid proteome.
• The fluid proteome exhibited tissue-specific expression, especially the fat body.
• Fluid proteome dynamics provided insights into FRT secretory mechanisms.

## Graphical Abstract

**Figure:**
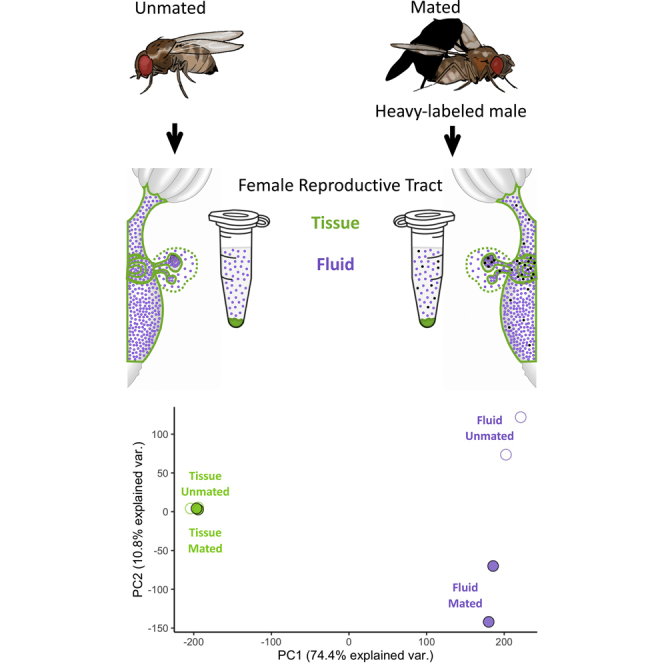

## In Brief

Fertility depends on coordinated postmating processes within the female reproductive tract (FRT), including ejaculate–FRT protein interactions that regulate sperm motility, storage, and modification. Semiquantitative proteomics was utilized to characterize the FRT tissue and, separately, luminal fluid, before and after mating. The dynamic mating-induced changes in the FRT fluid inform our understanding of FRT secretory mechanisms and ejaculate–female interactions that regulate fertility. Our study highlights the utility of applying proteomic approaches to characterize the extracellular FRT environment.

Ejaculate–female interactions within the female reproductive tract (FRT) mediate processes critical to fertility. For example, interactions among sperm, seminal fluid, FRT membranes, and the luminal FRT fluid influence sperm storage and survival, including postejaculatory modifications to sperm (1, 2, 3, 4, 5, 6). In particular, the molecular composition, viscosity, pH, and fluid dynamics of the FRT environment have been associated with sperm motility and viability (2, 4, 7, 8). The FRT fluid has also been found to have diverse enzymatic capacity, including the proteolytic processing of copulatory plugs and the modification or degradation of seminal fluid proteins (SFPs) (9, 10, 11, 12). These female contributions to ejaculate–female interactions may influence the mechanisms of postcopulatory sexual selection that bias the fertilization success of sperm from competing males ((13, 14, 15), reviewed in (16, 17, 18, 19, 20, 21)). As a result, the components of ejaculate–female interactions are likely to evolve rapidly and contribute to postmating, prezygotic reproductive barriers (22, 23, 24). Our understanding of the molecular basis of these interactions is heavily biased toward male contributions because of the widespread applications of proteomics to the study of ejaculate composition, including sperm (*e.g.*, insects (25, 26, 27, 28) and mammals (29, 30, 31, 32)) and SFPs (*e.g.*, insects (26, 33, 34, 35, 36, 37), birds (38, 39), and mammals (40)). Although there is increasing evidence supporting the importance of FRT fluid proteins to fertility, their identity, expression, regulation, and functionality have received limited attention. Ultimately, it is essential to investigate the female-derived molecules that participate in the ejaculate–female interactions to achieve a system-level characterization of reproductive processes.

The FRT fluid composition has primarily been examined in mammals, particularly those of relevance to human fertility and agriculture. These studies have shown that the FRT fluid predominantly comprised secretions from the FRT epithelium and can be highly heterogenous, differing in regional composition throughout the FRT (41, 42, 43, 44, 45, 46, 47, 48). Protein composition has also been shown to vary temporally across stages of the estrous cycle and pregnancy, as well as in response to mating and the presence of sperm and SFPs (41, 49, 50). Among the most commonly identified proteins are the glycoproteins, which likely interact with sperm (41, 42, 51), as well as protease inhibitors, immunoglobulins, and growth factors (41). Exosomes and other vesicles or microcarriers, which transport molecular cargo, are also a common feature in the FRT fluid and can fuse with sperm and potentially modify their composition (52, 53, 54). The potential role of fluid proteins in sperm modification is further supported by *in vitro* associations between oviductal fluid proteins and sperm (55). Although the specific functions of the female proteins associated with sperm have yet to be fully elucidated, interactions between the FRT extracellular vesicles and the sperm membrane are integral to proper capacitation and activation (56, 57).

Insects provide powerful systems to characterize the origin, composition, and function of the FRT fluid due to the discrete exocrine glands and the diversity of glandular secretion roles in reproduction (58, 59). The FRT glands are hypothesized to contribute to the FRT fluid primarily through merocrine secretion (*i.e.*, secretion of specific proteins *via* exocytosis) (59, 60). Although mechanisms of secretion in the FRT are not well established, apocrine or holocrine secretion (*i.e.*, secretion of some or all of the cytoplasmic contents of the cell) are also likely to contribute, as found in the male reproductive tract (61). Proteomic analysis of FRT tissues has been conducted in taxonomically diverse organisms (37, 62, 63, 64, 65), whereas proteomic analyses of FRT fluid have thus far been more restricted. Amongst insects, these analyses have been limited to social hymenopteran species where they have revealed that FRT secretions contribute to sperm viability over long-term storage, which can last up to several decades (66, 67). In the honey bee (*Apis mellifera*), spermathecal fluid proteins were found to be enriched for glycolysis and antioxidant defense (68) and are hypothesized to support an increased metabolic rate of sperm in storage (69). In addition, a recent comparison of spermathecal fluid proteome from queens across hives of varying health found that abundance of spermathecal fluid proteins was correlated with sperm viability (70). In the leafcutter ant (*Atta colombica*), spermathecal fluid proteins were enriched for proteolytic and oxidation-reductase activity (71). Although specific functions have yet to be determined for FRT fluid proteins, it is hypothesized that they impact sperm viability and may influence competition between the ejaculates of rival males (68, 71). In addition, a recent comparative analysis of postmating changes in the FRT tissue proteome between sibling *Drosophila* species (*simulans* and *mauritiana*), in which conspecific sperm precedence contributes to reproductive barriers (72, 73), identified widespread differences in the abundance of putatively secreted proteins, including an enrichment of proteases that may contribute to species-specific differences in the ejaculate–female interactions (62). Together, these studies suggest that the FRT produces a selective extracellular environment containing proteins that interact with the ejaculate in a manner that is likely to influence postcopulatory processes and may contribute to postmating, prezygotic reproductive isolation (23, 24).

In *Drosophila melanogaster*, the FRT fluid has not yet been characterized. However, several lines of evidence support the involvement of the FRT fluid in postcopulatory processes. First, studies of mutant lines lacking the primary FRT glandular tissues, the spermathecae and the parovaria, revealed that these tissues are necessary for proper sperm storage (74, 75, 76, 77). Second, the FRT glands have been shown to secrete proteins through both exocytotic pathways and noncanonical secretory pathways (77), and the other FRT tissues have histological indications of secretory capabilities (65, 78). Third, immunity proteins, vesicles, and neuromodulators have been visualized in the extracellular FRT and display regional and temporal variation in abundance (79, 80, 81). Fourth, FRT expressed genes that coevolve with SFPs in the well-studied sex peptide pathway are necessary for the regulation of female remating latency and stimulation of oogenesis and oviposition, further supporting the interaction of female secretions with the ejaculate (82). Finally, we recently conducted a comprehensive transcriptomic characterization of premating and postmating gene expression in the *D. melanogaster* lower FRT, which revealed that all 5 FRT tissues have enriched expression of tissue-specific secreted gene products (83). These tissue-specific secreted gene products also tend to evolve rapidly, as might be expected of proteins interacting and coevolving with the ejaculate (83). This comprehensive FRT expression dataset provides a foundation for investigations of the proteomic contents of the extracellular FRT environment.

Here, we characterize the proteome of the *D. melanogaster* lower FRT tissue and the fluid therein and analyze their compositional changes after mating. Our analyses demonstrate that the FRT fluid proteome (1) is complex and enriched for metabolic proteins, (2) is encoded by genes with more restricted, tissue-enriched patterns of expression, particularly in the FRT-associated fat body, and (3) experiences widespread postmating compositional changes relative to the FRT tissue. These novel insights regarding the dynamic changes of the extracellular FRT fluid provide the basis for future studies of specific ejaculate–female interactions that contribute to postmating processes essential to fertility and potentially influence competitive fertilization success and the maintenance of species boundaries.

## Experimental Procedures

### Fly Maintenance and Mating

*D. melanogaster* WT LH_M_ strain was maintained at RT (∼23 °C), with a natural light cycle on a standard yeast, cornmeal, agar, and molasses media. Experimental females were collected within 14 h of eclosion and matured in vials of 10 to 15 flies with media supplemented with live yeast for 3 to 8 days. For mated samples, 15 to 20 males were added to a vial of females, and dissections were conducted 6 h after mating (±1 h after the introduction of males). This time point was selected because that is the time of maximal postmating transcriptomic response (64, 83). Note that all males were isotopically labeled (see below) so that female- and male-derived proteins could be distinguished.

### Dissections and Sample Isolation

Lower FRTs (*i.e.*, bursa, oviduct, parovaria, spermathecae, seminal receptacle, and tightly associated fat bodies) were dissected from etherized females in 1× PBS. Dissections were conducted to prevent any contamination from ovulated eggs or nearby gut tissue. For postmating samples, successful mating was visually confirmed by the presence of sperm in the FRT. FRTs from at least 150 females per replicate were dissected, rinsed in another PBS drop, and transferred into a 1.5-ml Eppendorf tube with 50-μl PBS. FRT fluid was isolated from the FRT tissue using an adaptation of methods used to collect FRT contents to identify the ejaculate proteins transferred during mating (34). Specifically, to minimize tissue damage, FRTs were centrifuged at 7500 rpm for 10 min to separate the tissue and fluid. The approximately 100 μl of the supernatant (enriched for the FRT fluid) was transferred and combined with 10 μl of 1 M Hepes + 2% SDS and 5% tris(2-carboxyethyl)phosphine (TCEP), heated for 15 min, and stored at −80 °C. The remaining FRT tissues were solubilized in 100 μl of 1 M Hepes with 2% SDS and 5% TCEP. The tissues samples were heated at 95 °C and homogenized with a pellet pestle until completely solubilized and stored at −80 °C. In total, we collected two replicates for each of the following: (1) FRT fluid from unmated females, (2) FRT fluid from mated females, (3) FRT tissue from unmated females, and (4) FRT tissue from mated females.

### MS

Proteomic analyses were conducted by the Cambridge Centre for Proteomics following standard protocols. The protein samples were quantified with an EZQ Protein Quantitation kit (Thermo Fisher Scientific), as per the manufacturer's instructions. For each sample, 15 μg of protein was separated by size on a 1.5-mm 12% SDS-PAGE gel stained with colloidal Coomassie dye and divided into ten slices (supplemental Fig. S1). The FRT tissue and fluid samples were run in a standardized fashion. Gel fractions for each sample were reduced (DTT), alkylated (iodoacetamide), trypsin-digested (overnight at 37 °C), and eluted (0.1% formic acid). The samples were then analyzed with a Dionex UltiMate 300 rapid separation liquid chromatography nanoUPLC system (Thermo Fisher Scientific) coupled with a Q Exactive Orbitrap mass spectrometer (Thermo Fisher Scientific). Peptides in fractions were first filtered through a precolumn (PepMap 100 C18, 5-μm particle, 100 Å pore, 300 μm × 5 mm, Thermo Fisher Scientific) for 3 min at 10 μl/min with 0.1% formic acid. The peptides were then eluted to the analytical reverse-phase nano EASY-Spray column (PepMap C18, 2 mm particle, 100 Å pore, 75 mm × 50 cm, Thermo Fisher Scientific) and separated by C18 reverse-phase chromatography at 300 nl/min with 0.1% formic acid with a gradient of 1.6% to 32% acetonitrile over 90 min (total run time 120 min, including column wash and equilibration). The eluted peptide (transferred *via* EASY-Spray source; Thermo Fisher Scientific) ion *m/z* values were measured *via* the mass spectrometer (between 380 and 1500 Da, 70,000 resolution). Data-dependent MS/MS scans (MS1 followed by MS2, top 20) isolated and fragmented precursor ions by collision-induced dissociation (32.5%, normalized collision energy) and analyzed (resolution of 35,000) in the linear ion trap within a 60 s ± 10 ppm dynamic exclusion window (ions were also excluded if they were singly charged or had unassigned charge state).

### Protein Identification

The resulting peptide mass spectra were identified with PEAKS Studio X (Bioinformatics Solutions Inc.). Identification of unlabeled (*i.e.*, female-derived) proteins was based upon an analysis using the *D. melanogaster* reference genome protein annotation (r6.32) (84), including only the longest protein isoform of each gene (13,968 entries), appended with the cRAP v 1.0 contaminant database (thegpm.org). We note that restricting the search database to the longest isoforms precludes the evaluation of alternative splicing. Search parameters allowed for semispecific digestion with three missed tryptic cleavages as well as parent monoisotopic mass error of 15.0 ppm and fragment ion mass tolerance of 0.5 Da. Post-translational modifications included carbamidomethylation (cysteine; fixed), oxidation (methionine; variable), and deamidation (glutamine and arginine; variable). The samples contained a total of 2.14 million spectra resulting in the identification of 876,082 peptide–spectrum matches (PSMs). PSMs were included if their −10logP ≥ 30 (total false discovery rate [FDR] <0.05 estimated with a decoy-fusion approach (85)) had a PTM A score >100, and a *de novo* identified score ≥50. Protein inclusion required a −10logP ≥ 20 and identification by at least two unique peptides, at least two spectral hits, and a spectral area greater than zero in either all tissue replicates or all fluid replicates. These criteria resulted in a total of 1840 identified proteins (supplemental Table S1). MS data are available *via* the ProteomeXchange Consortium (PRIDE partner repository, PXD025085).

### Protein Abundance Quantitation

Differential abundance analyses were conducted using the PEAKS quantitation software allowing for comparisons of abundance estimates to be fine-tuned on the direct comparison of the spectra (86, 87). Specifically, estimation of spectral area allowed for a mass error tolerance of 20.0 ppm and retention time shift tolerance of 6.0 min and was normalized to the sum of the total peak area to account for background intensity differences across the samples. Separate analyses were conducted to identify differentially abundant proteins between the following: (1) tissue and fluid samples, including both mated and unmated samples (n = 1132 proteins), (2) unmated and mated tissue samples (n = 1558 proteins), and (3) unmated and mated fluid samples (n = 715 proteins). The number of proteins per comparison varies from the number of proteins identified because the quantitative analysis was dependent upon high-quality peak intensity estimates for all proteins. PEAKS Q significance values (approximately equivalent to −10log10 *p*-value) were converted to *p*-values and corrected for multiple comparisons with the Dunn–Bonferroni correction. Proteins were considered differentially abundant if the adjusted *p*-value was ≤0.05 (supplemental Table S1).

### Production of Double Auxotrophic Yeast and *Drosophila* Heavy Labeling

A double auxotrophic (Lys and Arg) mutant *Saccharomyces cerevisiae* strain was produced from the diploid yol058W/yol058W (BY4743 yeast deletion collection strain background SGD: S000005419), which is a diploid homozygous Arg deletant strain (arg1/arg1) that is also heterozygous for the Lys mutant (LYS2/lys2) (88). To obtain candidate arg1 lys2 strains, the parent diploid was sporulated using Simchen sporulation media at RT for 5 days, after which the culture was pelleted, washed, and stored in sterile water at 4 °C (89). A 5-μl aliquot of the sporulated yeast culture was mixed with 40 μl with 10% 2 mg/ml Zymolyase 100T and was incubated at 30 °C for 13 min and then spread onto yeast extract–peptone–dextrose (YPD) medium plates before tetrad dissection. Tetrads were dissected with a micromanipulator, and the germinated spores were grown as colonies for 2 to 3 days on YPD plates at 30 °C. For tetrads with four visible progenies, each colony was streaked onto YPD plates, regrown to confluence, and replicate plated onto synthetic complete media without Arg or Lys (Sunrise Science Products) to identify haploid strains auxotrophic in both amino acids. The haploid arg1 lys2 auxotrophic strain with the most robust growth rate was selected for subsequent use. This strain was grown following a standard 2-day incubation protocol in a liquid culture with synthetic complete media powder excluding Arg and Lys (Sunrise Science Products), supplemented with 85 mg/L each of isotopically labeled lysine (^13^C_6_^15^N_2_) and arginine (^13^C_6_^15^N_4_) (Cambridge Isotope Laboratories). At saturation (*A*_600_ 0.7–0.8), yeast was washed twice with sterile water, pelleted, and stored at −20 °C.

*D. melanogaster* embryos (fertilized eggs before hatching) were collected, washed to remove any yeast, and transferred (25 eggs/vial) to vials with 4 ml heavy-yeast media (modified from standard media with 0.8% low-melting temperature agarose, 15 g/100 ml sucrose, and 3 g/100 ml heavy-labeled yeast) on top of a 4-ml layer of 2.5% agar to prevent media from drying out. The media were further supplemented with a small pellet of labeled yeast. Within 14 h of eclosion, flies were collected, sexed, and transferred to a new vial with heavy-labeled yeast and agar media. Males were aged at least 5 days and had mated at least once before this experiment.

### Isotopic Labeling Efficiency

Labeling efficiency was determined using whole-fly samples from five females and five males. Flies were flash-frozen and solubilized in 2× Laemmli buffer with the TCEP reducing agent with alternating cycles of homogenization with a pellet pestle and heating at 95 °C. The samples were then centrifuged at 17,500*g* for 3 min to remove any insoluble material. The samples were prepared for MS analysis as described above. Each sample was trypsin-digested, reconstituted in 0.5% formic acid, and analyzed on an Orbitrap Fusion Tribrid (Thermo Fisher Scientific) mass spectrometer with a Nanospray Flex Ion Source coupled with a Dionex UltiMate 3000 RSLCnano system (Thermo Fisher Scientific). Peptide filtering, elution, and separation with equivalent columns and settings as described above. The Orbitrap Fusion was operated in a positive ion mode (spray voltage 1.6 kV and source temperature 275 C) with data-dependent acquisition analysis (1.6 m/z quadrupole isolation, 10,000 threshold ion count, and 30% normalized collision energy) and a 50 s ± 10 ppm dynamic exclusion window using Xcalibur 2.0 operation software (Thermo Fisher Scientific).

The spectra were then analyzed using PEAKS studio X (Bioinformatics Solutions Inc.) with settings for unlabeled protein identification as described above with the addition of variable modifications for SILAC Arg (13C_6_ and 15N_4_), Lys (13C_6_ and 15N_2_), and Arg conversion to proline (R to P [13C_5_] and R to P [13C_5_-15N_1_]). PSMs were included if they achieved a −10logP ≥ 35 (total FDR <0.05). As we were not concerned about ascertaining the specific location of PTMs, no threshold PTM localization A score was specified. This analysis resulted in 5628 PSMs, 91.3% of which (5140/5628) were labeled (supplemental Table S2). We note that this is an estimate for the whole body but do not have any reason to expect that it would not be an accurate proxy for labeling of sperm proteins and SFPs. Furthermore, we cannot entirely preclude the identification of some male-contributed proteins because of partial labeling but note that no male-specific (*i.e.*, no detectable gene expression in the FRT (83)) sperm proteins or SFPs were identified in mated samples. MS data are available *via* the ProteomeXchange Consortium (PRIDE partner repository, PXD025072).

### Experimental Design and Statistical Rationale

In total, we analyzed four biological replicates of the FRT tissue and fluid (two from unmated and two from mated females). This design provided robust replication (n = 4) for distinguishing proteomic differences between the tissue and fluid proteomes, as well as sufficient replication (n = 2 for tissue and fluid, respectively) for quantitative analyses of postmating proteomic changes. Robust thresholds were applied for both peptide and protein identification, including the establishment of FDRs estimated using a decoy-fusion approach (85). Accuracy of quantitative estimates was ensured using stringent mass tolerance and retention time shift thresholds. Differential abundance was calculated based on the spectral area using PEAKS quantitation software with stringent *p* value cutoffs after correction for multiple testing (86, 87). We mated females to heavy-labeled *Drosophila*, produced by rearing larvae on media with double auxotrophic (arg1 lys2) yeast supplemented with both heavy Arg and Lys isotopes, thus significantly improving the likelihood that all male peptides were distinguishable by labeled amino acids after tryptic digestion. In addition, independent MS/MS experiments were conducted to confirm the efficiency of heavy labeling (91.3%; see above).

### Functional Annotation and Statistical Analysis

Functional enrichment was conducted with clusterProfiler (90). Categories were considered enriched if the Benjamini–Hochberg–adjusted *p*-value was <0.01. Functional enrichment analyses for proteins identified in the FRT tissues or fluid were conducted using the 8337 genes of the FRT transcriptome (83), whereas differentially abundant proteins were compared with the background of proteins identified in the combined tissue and fluid proteome (1840 proteins). Significant enrichments had an adjusted *p*-value of < 0.05. Proteins differentially abundant between the tissue and fluid were further analyzed with STRING database (version 11) using high confidence (>0.7) support for protein interactions and functional enrichment of proteins within these networks (91). Comparison of the representation of exosomes and vesicles between the fluid and tissue datasets was based on genes identified in the ExoCarta database (92). Protein length was obtained from FlyBase (84), and codon bias (ENCprime) was determined using coRdon (93). Differences in protein length or codon bias between protein groups were determined using a nonparametric Kruskal–Wallis test.

To evaluate relationships among samples, the spectral area was log2-transformed and median-normalized. Proteome reproducibility among samples was determined with a Pearson's correlation and visualized with a complete-linkage hierarchical clustering heatmap (94). Relationships among samples were also analyzed with a principal component (PC) analysis. Spearman's correlation was used to evaluate the relationship between protein abundance and gene expression. To standardize comparisons between fluid and tissue proteomes, a sampling with replacement approach was used to account for sample size differences. Spearman's correlation was also used to compare log2FC between the FRT tissue or fluid proteome and individual FRT tissues. Directional biases in changes of protein abundance were analyzed using a weighted binomial test. A chi-square test was used to compare the proportions of proteins between categories (*i.e.*, tissue *versus* fluid proteins). We used a nonparametric Kruskal–Wallis test to compare the differences in the mean protein abundance or changes in protein abundance. Variance in postmating log2 fold change was analyzed with a Levene's test. All remaining data visualizations were produced with ggplot2 (95). All analyses were conducted in R 3.6 (96) and are available on GitHub at https://github.com/CEMcDonoughGoldstein/FRT.TissueFluid.Proteome.

## Results

### FRT Tissue and Fluid Proteomes Have Distinct Characteristics

We characterized the FRT tissue and fluid proteomes of unmated and mated females after isolating the fluid contents of the FRT lumen. Females were mated to heavy-labeled males so that postmating changes in FRT protein abundance could be distinguished from male-derived proteins transferred in the ejaculate. The distinct characteristics of the FRT tissue and fluid proteomes were initially apparent in their 1D SDS-PAGE banding patterns, which indicated that their protein composition was substantially different (supplemental Fig. S1, *A* and *B*). The four tissue and four fluid replicates, respectively, were highly quantitatively reproducible (all r ≥ 0.8) and formed discrete clusters based on compositional similarity (supplemental Fig. S1*C*), further supporting the distinct nature of these samples. In the following analyses, we investigate the proteomic differences between the FRT tissue and FRT fluid samples.

**Figure 1:**
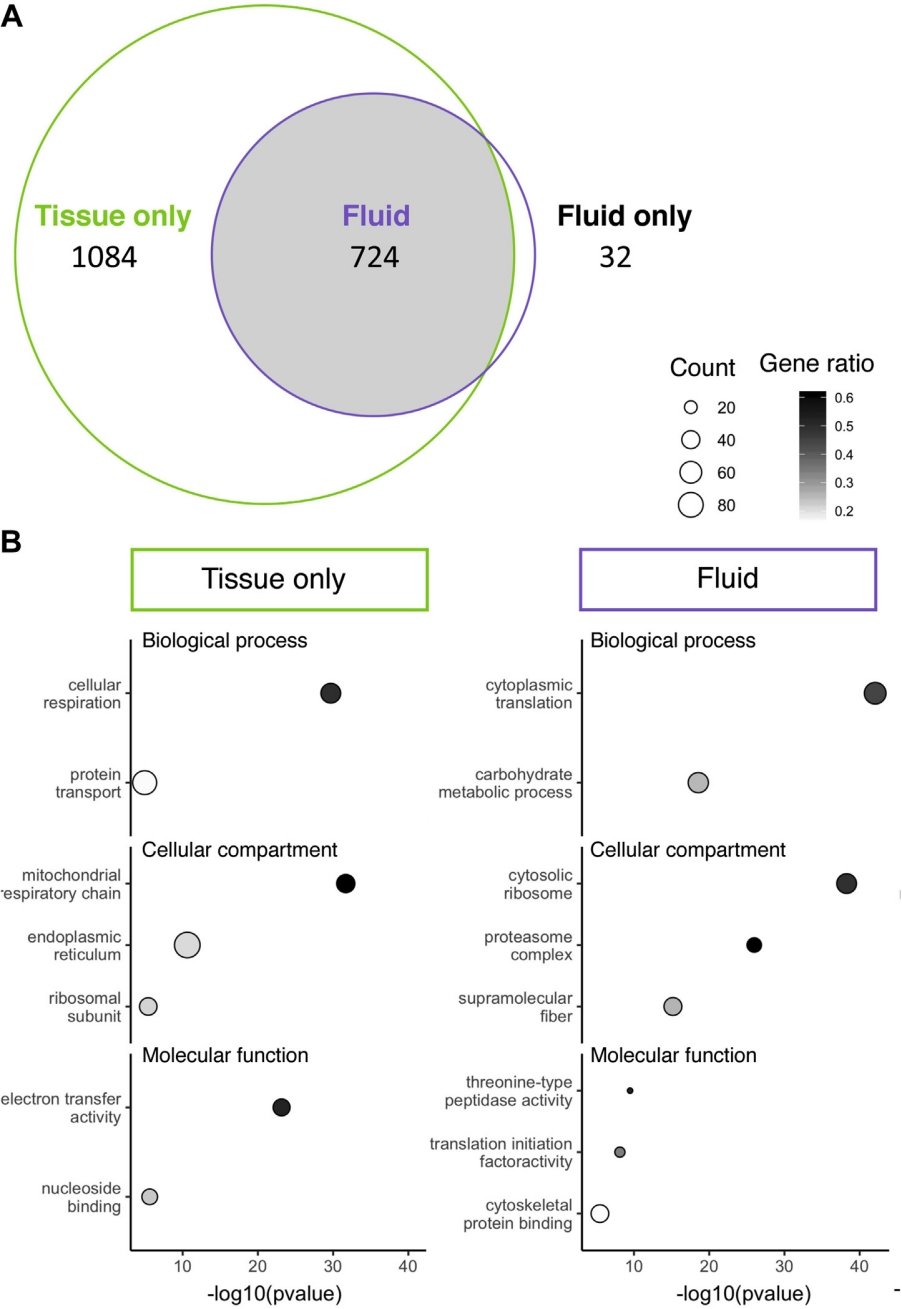
**Protein identification between the tissue and fluid**. *A*, Venn diagram of proteins identified in the tissue (1808 proteins) and the fluid (756 proteins). The tissue proteome has twice as many proteins as the fluid, with 1084 proteins exclusively identified in the tissue. The fluid is almost a complete subset of the tissue with only 4.6% (32/756) of proteins uniquely identified in the fluid. *B*, representative gene ontology functional enrichments for tissue-only and fluid proteins.

The FRT tissue proteome was significantly more complex than the FRT fluid proteome, containing over twice as many proteins (1808 proteins in the tissue *versus* 756 proteins in the fluid). Consistent with the expectation that the fluid proteins should also be present in the tissues that produce them, the fluid proteome was almost entirely a subset of the tissue proteome (95.8%; 724 of 756 proteins; Fig. 1*A*). In addition, fluid proteins identified in the tissue exhibited significantly higher abundance levels than the remainder of the proteins in the tissue proteome (supplemental Fig. S2, *A* and *B*; Kruskal–Wallis χ^2^ = 296.9, *p* < 0.001). To account for the possibility that contaminating tissue material contributed to the fluid proteome, we evaluated the depth of coverage between the tissue and fluid samples. Although there were less than half as many PSMs in the fluid samples (tissue average: 18,633 ± 552 and fluid average: 8750 ± 1142), a comparable depth of protein coverage was achieved (mean unique PSMs per protein, tissue average: 10.34 ± 0.31, and fluid average: 11.64 ± 1.52). Thus, the fluid had a greater number of unique PSMs per protein relative to the total number of PSMs identified. This pattern supports concentrated proteomic coverage within a specific subset of proteins found in the FRT fluid.

The distinct nature of the tissue and fluid proteomes was further supported by the differences in functional enrichments (Fig. 1*B*). Tissue-only proteins were significantly enriched for gene ontology (GO) annotations associated with intracellular components, such as the mitochondria, and essential cellular functions of respiration and protein transport (Fig. 1*B*; supplemental Table S3). In contrast, fluid proteins were enriched for GO annotations associated with enzymatic breakdown of proteins (Fig .1*B*; supplemental Table S3), which was consistent with the previously described proteolytic activity in the insect FRT fluid (9, 10, 12). The small number (n = 32) of fluid-only proteins was also enriched for the regulation of proteolytic activity, thus providing further support for the enzymatic activity in the FRT fluid (supplemental Table S3). Fluid proteins were also enriched for functions relating to cytoplasmic translation and association with cytoskeleton. We note that major cytoplasmic protein components, such as translational machinery, have been observed as a result of holocrine or apocrine secretion (*e.g.*, (97)). Although the fluid proteome exhibited a significant underrepresentation of proteins encoded by genes with secretion signals relative to the tissue (lower-tail binomial cumulative probability test, *p* = 0.01; 11.8% of fluid proteins, and 17.5% of tissue-only proteins), an enrichment of exosome- or vesicle-associated annotations was observed (upper-tail binomial cumulative probability test, *p* < 0.001; 30.0% of fluid proteins, and 8.3% of tissue-only proteins had exosome or vesicle annotations). Based on these complementary pieces of evidence, we contend that our methodology successfully enriched for the FRT fluid and resulted in an informative proteome with respect to fluid composition.

### Tissue-Specific Expression of Fluid Proteins

**Figure 2:**
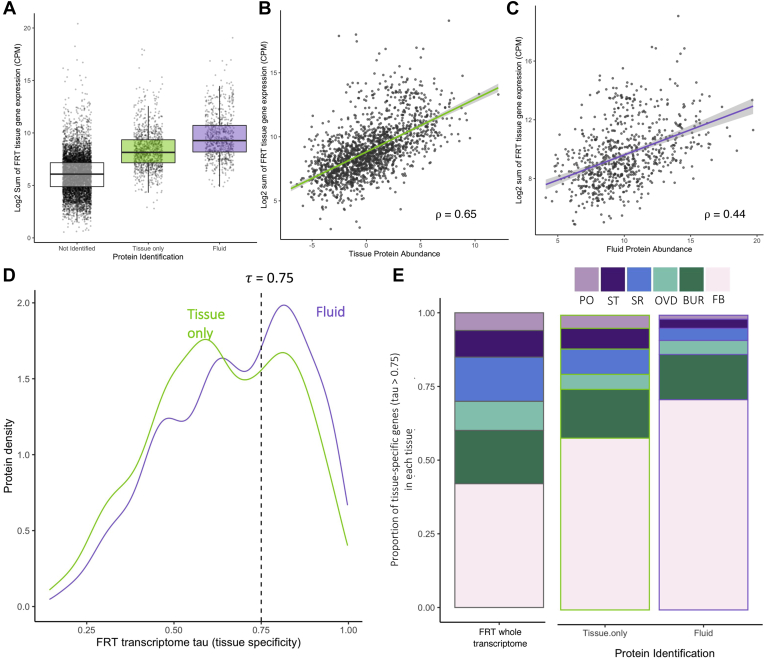
**Relationship of FRT tissue and fluid proteome to the FRT tissue transcriptome.***A*, genes encoding identified proteins tend to have higher expression. Protein abundance in the tissue (*B*) and fluid (*C*) is significantly correlated with the sum of gene expression across the FRT tissues. *D*, density distribution of proteins based on their tissue specificity, as measured by gene expression across the five FRT tissues, fat body, and whole female body. *E*, for proteins encoded by genes with tissue-specific expression, we investigated the proportion of genes expressed in each FRT tissue. The FRT-associated fat body has the greatest proportion of tissue-specific genes encoding both the tissue-only and fluid proteins. Background proportion of all the FRT-expressed genes with a tau >0.75 is shown for comparison. BUR, bursa; FB, FRT-associated fat body; FRT, female reproductive tract; OVD, oviduct; PO, parovaria; SR, seminal receptacle; ST, spermatheca.

To evaluate how the FRT tissues (*i.e.*, bursa, oviduct, seminal receptacle, spermathecae, and parovaria) and FRT-associated fat body may contribute to the fluid composition, we compared the fluid proteome with our recent comprehensive analysis of gene expression across the FRT tissues (83). First, we noted that nearly all the proteins identified in the tissue and fluid proteomes were identified in the FRT tissue transcriptome (95.3% of proteins; representing 22.1% of all genes expressed in the FRT transcriptome). Second, we found that genes encoding proteins identified in both the FRT tissue and fluid proteomes had significantly higher FRT expression than genes not identified in the FRT proteome (Kruskal–Wallis χ^2^ = 2215.5, *p* < 0.001; Fig. 2*A*). In addition, FRT gene expression of fluid proteins was significantly greater than that of tissue-only proteins (Kruskal–Wallis χ^2^ = 157.36, *p* < 0.001). Protein abundances in both the tissue and fluid, respectively, were also significantly correlated with gene expression across the FRT tissues (tissue ρ = 0.65, *p* < 0.001.; fluid ρ = 0.45, *p* < 0.001; Fig. 2, *B* and *C*). We note, however, that the relationship was more robust between the tissue proteome and FRT transcriptome, consistent with a more direct relationship between gene expression and intracellular protein abundance. The weaker relationship with the fluid proteome may be due, in part, to the impact of secretory mechanisms, which further decouples extracellular protein composition from FRT gene expression.

Third, we evaluated the representation of tissue-specific gene products in the proteome. Using the FRT tissue transcriptome (83), we initially compared the distribution of fluid and tissue-only proteomes based on previously calculated estimates of specificity of gene expression (using the statistic tau, where tau = 0 indicates equal expression in all tissues and tau = 1 indicates exclusive expression in a single tissue). We found that genes encoding the fluid proteome had a distribution significantly biased toward tissue-specific expression compared with those encoding the tissue-only proteome (Kolmogorov–Smirnov D = 0.11, *p* < 0.001; Fig. 2*D*). We then determined which tissues were represented among the set of genes with tissue-specific expression (*i.e.*, tau >0.75). For both the tissue and fluid proteomes, the proportions of specific genes from each of the five FRT tissues and the fat body were significantly different from the proportions across the FRT transcriptome (tissue: χ^2^ = 858.5, *p* < 0.001 and fluid: χ^2^ = 1939.2, *p* < 0.001; Fig. 2*E*). In particular, the tissue-only proteome was derived from a significantly higher proportion of fat body–specific genes (χ^2^ = 33.5, adjusted *p* < 0.001) and lower proportion of seminal receptacle–specific genes (χ^2^ = 9.6, adjusted *p* =0.02) than all FRT tissue-specific genes. This pattern was even stronger in the fluid proteome that had an even greater overrepresentation of fat body–specific genes (χ^2^ = 20.0, adjusted *p* < 0.001) and underrepresentation of seminal receptacle–specific genes (χ^2^ = 7.0, adjusted *p* = 0.05) than the tissue-only proteins (Fig. 2*E*). Thus, in contrast to the FRT-associated fat body, the glandular spermatheca and parovaria appear to contribute a relatively small number of tissue-specific products to the FRT fluid. This pattern supports the hypothesis that the tightly associated FRT fat body, which was found to have a distinct transcriptomic profile relative to the remainder of FRT tissues, contributes a substantive number of proteins to the FRT lumen (83).

### Fluid-Biased Proteins Are Enriched for Metabolic Functions

**Figure 3:**
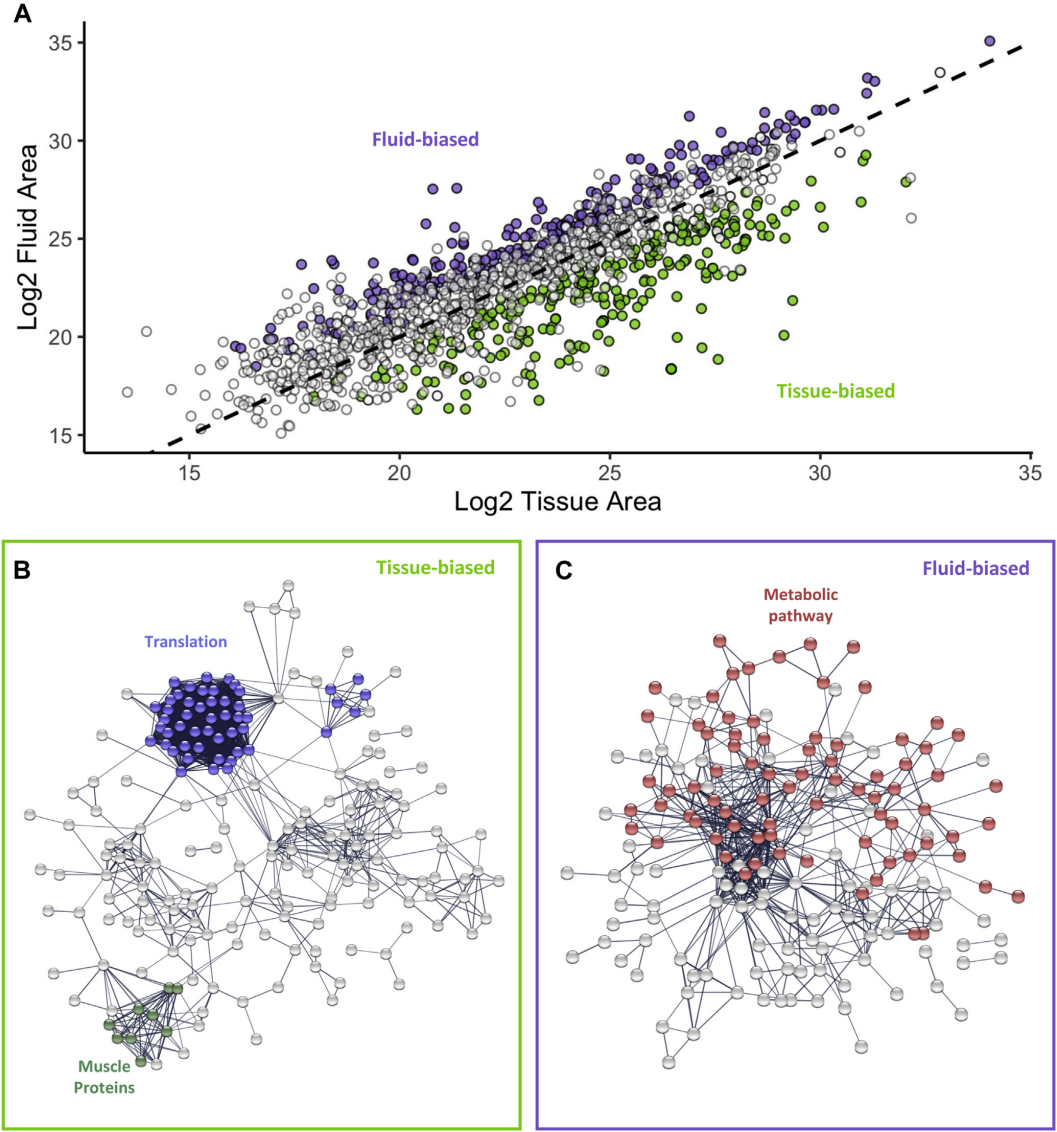
**Biased abundance of fluid proteins.***A*, protein abundance (Log2 peak area) of proteins detected in both tissue and fluid was significantly correlated (r = 0.80, *p* < 0.001). The *dashed line* represents 1:1 abundance (or perfect positive correlation between protein abundances in the tissue and fluid). However, there were significant differences in protein abundance with similar numbers of proteins that had biased abundance in the tissue (245; *green*) or the fluid (236; *purple*). STRING network analysis of biased proteins in the tissue (*B*) and fluid (*C*). The tissue-biased proteins were enriched for translation, including a dense network of ribosomal proteins (*blue*). There was also a densely connected network of muscle proteins (*green*), although it was not significantly enriched. Fluid-biased proteins were enriched for KEGG metabolic pathway annotation (*red*).

To investigate the functional composition of proteins highly represented in the fluid, we compared protein abundance between the tissue and fluid proteomes. Overall, the abundance of proteins identified in both the FRT tissue and fluid were significantly correlated (r = 0.80, *p* < 0.001, Fig. 3*A*). However, there were a substantial number of significant protein abundance differences (adjusted *p* < 0.05; 236 fluid-biased and 245 tissue-biased). We found that the tissue-biased proteins were part of a protein network with significantly more interactions than expected (1402 edges observed, 1075 edges expected, and PPI enrichment *p* < 0.001), including hubs of proteins with intracellular functions such as ribosomal (cytoplasmic translation, GO:0002181, *p* < 0.001) and muscle proteins (KW-0514, *p* = 0.025; Fig. 3*B*; supplemental Table S4). Fluid-biased proteins were also part of networks significantly more interconnected than expected (609 edges observed, 323 edges expected, and PPI enrichment *p* < 0.001; Fig. 3*C*) but were enriched for metabolic processes (GO:0005975, *p* = 0.006), specifically glycolysis/gluconeogenesis (map00010, *p* = 0.013). The enrichment of metabolic pathways in the FRT fluid was consistent with previous insect FRT fluid analyses (68, 71) and suggests the active secretion of a subset of proteins from FRT tissues to form the fluid.

### Dynamic Postmating Changes in FRT Fluid Proteome Composition

**Figure 4:**
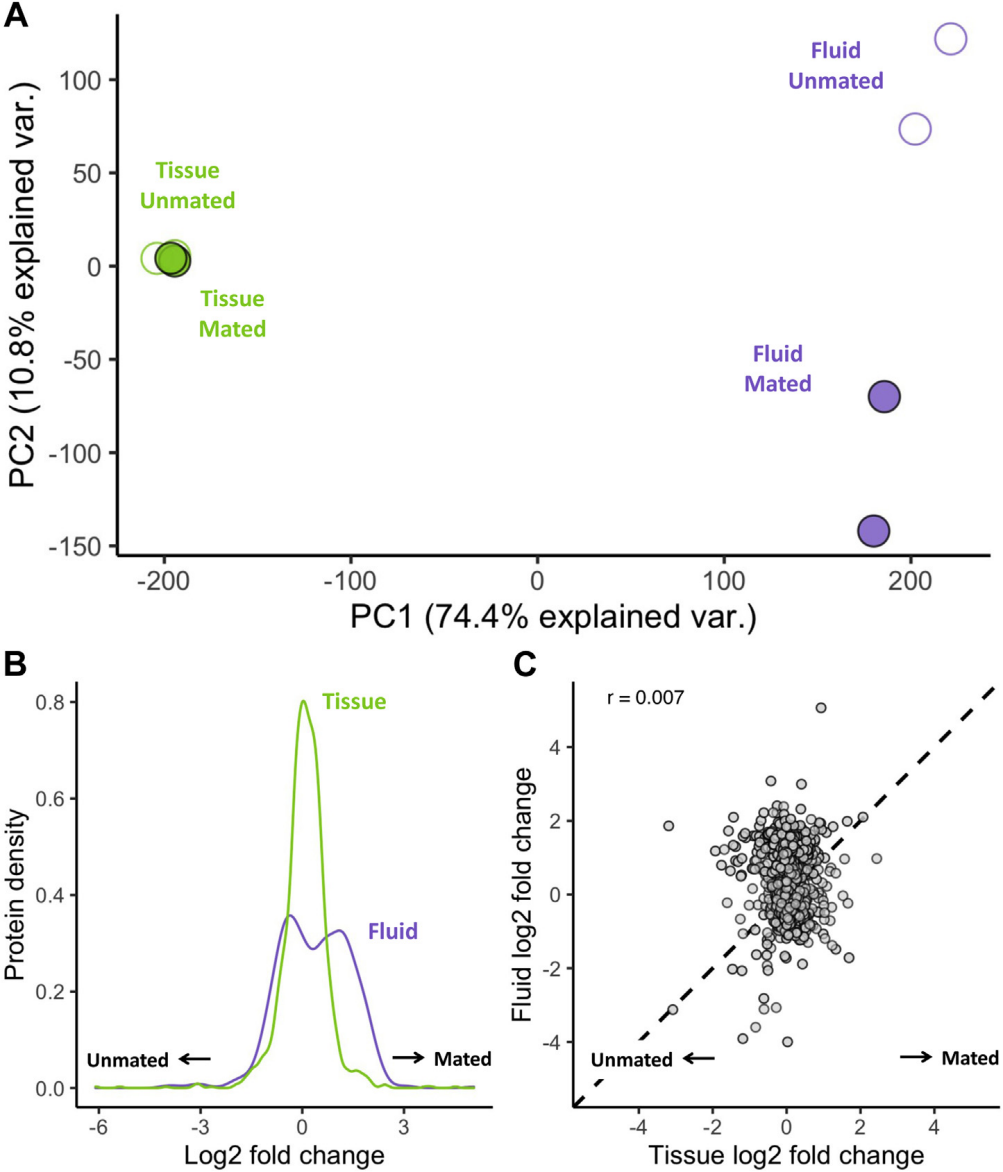
**Postmating changes in protein abundance were greater in FRT fluid.***A*, PCA of FRT tissue and fluid proteomes. The first principal component explained 74% of the variation between samples and separated tissue (*green*) from fluid (*purple*) samples. The second principal component explained 11% of variation and separates unmated (*open circles*) and 6-h postmating (mated; *closed circles*) fluid samples. *B*, density plot of log2 fold changes in abundance in the fluid (*purple*) and tissue (*green*). Fluid proteins had a significantly greater variance in log2 fold change than tissue proteins (*p* < 0.001). *C*, the log2 fold change in proteins was not correlated (r = 0.006, *p* = 0.86) between the tissue and fluid. The *dashed line* represents a 1:1 log2 fold change. FRT, female reproductive tract; PCA, principal component analysis.

To investigate how the FRT tissue and fluid proteomes changed in response to mating, we compared samples from unmated females with those 6 h after mating, the time of maximal postmating transcriptomic response (64, 83). A PC analysis revealed that more than 85% of the variation among samples is explained by the first two PCs. PC1 captured 74.4% of the variation and separated tissue and fluid samples. PC2 captured 10.8% of the variation and separated unmated and mated fluid samples, but not the corresponding tissue samples (Fig. 4*A*). Thus, a major axis of variation was due to postmating changes in fluid composition. To confirm this pattern, we compared the distribution of protein abundance changes and found that the fluid proteome exhibited a significantly greater variance in postmating response relative to the tissue proteome (Levene's F = 283.5, *p* < 0.001; Fig. 4*B*). The difference in postmating response was further reflected by the fact that 37% of the fluid proteome exhibited an absolute LogFC >1 compared with only 9% of the tissue proteome. We next compared protein abundance changes between postmating tissue and fluid proteomes and found no correlation (r < 0.007, *p* = 0.86, Fig. 4*C*). Together, these analyses indicate that the FRT fluid exhibits a pronounced postmating response at 6 h that is distinct from the more limited changes in the FRT tissue.

**Figure 5:**
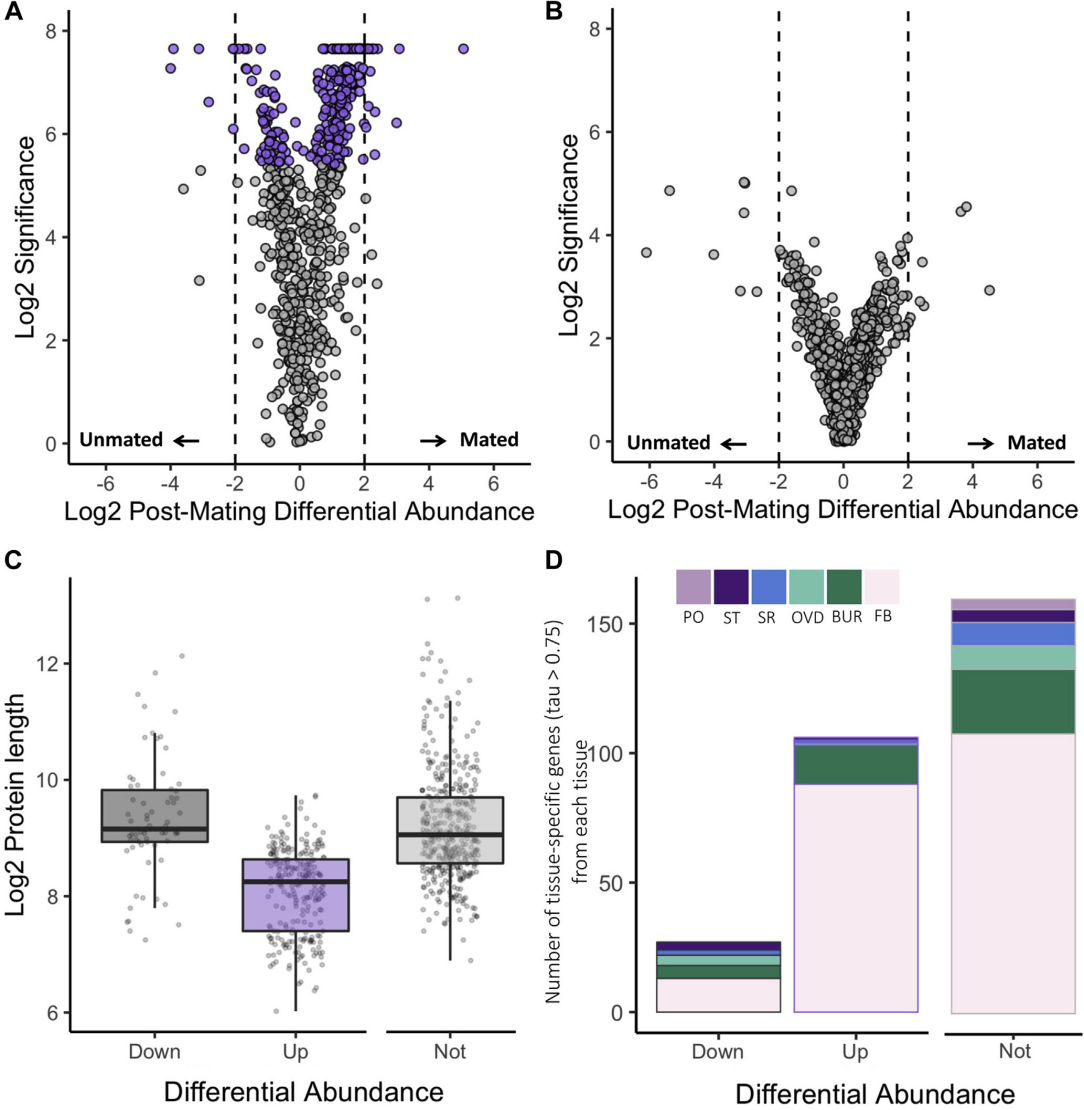
**Differences in postmating changes between FRT tissue and fluid.** Volcano plot of postmating change in abundance in the FRT (*A*) fluid and (*B*) tissue. 308 proteins were significantly differentially abundant in the fluid (*purple*), whereas no proteins were significantly differentially abundant in the tissue. Significance was based on PEAKS quantitative output and is equivalent to −10log10 *p*-value after multiple testing correction. *C*, proteins that increased in abundance after mating were significantly shorter in length than those that decreased in abundance or did not change significantly. *D*, for proteins with tissue-specific expression tau >0.75 we investigated, the proportion of tissues had maximum expression. FRT-associated fat body has the greatest proportion in both the tissue-only and fluid proteins. Background proportion of all the FRT-expressed genes with a tau >0.75 is shown for comparison. BUR, bursa; FB, FRT-associated fat body; FRT, female reproductive tract; OVD, oviduct; PO, parovaria; SR, seminal receptacle; ST, spermatheca.

We examined significant changes in response to mating and found that in total, 308 fluid proteins (40.7% of the total fluid proteome) exhibited significant changes in abundance (Fig. 5*A*), whereas no proteins in the tissue proteome were found to be significantly different (Fig. 5*B*). Among the differentially abundant fluid proteins, there were nearly four times as many proteins that increased in abundance (241 proteins) rather than decreased in abundance (67 proteins) after mating, an observation consistent with increased postmating secretory activity. We note that there was no significant enrichment for signal sequences among differentially abundant proteins (upper-tail binomial cumulative probability test, *p* = 0.92), suggesting that they may be secreted *via* alternative pathways. Proteins with greater postmating abundance had numerous significant functional enrichments relating to translation, similar to the enrichments observed in the whole fluid proteome (supplemental Table S3). There were no functional enrichments for the smaller set of proteins that exhibited decreased postmating abundance.

The minimal abundance changes in the FRT tissue proteome, relative to the fluid, may be explained by rapid translational replenishment of secreted proteins. To test this hypothesis, we examined whether fluid proteins might be more efficiently translated by assessing codon bias and length. On average, fluid proteins that increased in abundance postmating were significantly shorter than those that were not differentially abundant (Kruskal–Wallis χ^2^ = 8.58, adjusted *p* < 0.01) or decreased in abundance (Kruskal–Wallis χ^2^ = 19.43, adjusted *p* < 0.001; Fig. 5*C*). However, we observed no differences in codon bias between proteins that increased, decreased, or did not change in abundance after mating (Kruskal–Wallis χ^2^ = 0.56, *p* = 0.76; supplemental Fig. S3). Although further investigation is required, higher rates of translation among proteins that increase in abundance in the fluid proteome after mating may contribute to their rapid replenishment within FRT tissues.

### FRT Expression Dynamics of Mating Responsive Fluid Proteins

Finally, for differentially abundant fluid proteins, we examined underlying patterns of gene expression to determine if postmating fluid changes could be associated with particular FRT tissues (83). We found that the protein products of genes with fat body–specific expression were significantly overrepresented within fluid proteins that increased after mating compared with those that did not change (test of equal proportions, χ^2^ = 12.3, adjusted *p* = 0.003; Fig. 5*D*). We also compared protein abundance changes with gene expression changes at the same postmating time points (83). We observed a minimal relationship in the postmating changes between gene expression in any FRT tissue and fluid protein abundance (r = 0.15 ± 0.07). Although these relationships are marginal, we note that there was a significant positive correlation with the bursa, oviduct, and parovaria and a significant negative correlation with the FRT-associated fat body (supplemental Fig. S4). The lack of robust relationships in any of these comparisons suggests that postmating changes in fluid protein abundance are not directly related to postmating gene expression changes that occur up until this time point and are more likely related to secretory products already in place in an FRT poised to respond to mating.

## Discussion

A thorough understanding of the FRT, and especially the extracellular luminal environment, is critical for advancing mechanistic insights into infertility, postcopulatory sexual selection, and postmating prezygotic reproductive isolation (98). Across all animal taxa, FRT secretions are likely necessary for SFP modifications, which in turn regulate female postmating responses (82, 99, 100), postejaculatory modifications to sperm (6), and the processing, including degradation and ejection, of spermatophores and mating plugs (101). The FRT is also the critical selective environment underlying the dramatic diversification of male ejaculate components such as sperm (102). It is unsurprising, therefore, that the FRT is also known to be evolutionarily dynamic (3, 5, 19, 21). Diversification in FRT expression and postmating responses have been demonstrated between multiple *Drosophila* species pairs (62, 103, 104) and, more recently, within *Drosophila pseudoobscura* in response to experimental variation in the intensity of sexual selection (105). Although similar investigations of the *D. melanogaster* FRT have not been conducted, variation in female whole-body or body-segment gene expression and postmating response has been demonstrated between populations or selection lines (106, 107, 108). Despite accumulating evidence that variation in FRT secretions can dramatically impact male reproductive success, the application of ‘omic’ technologies to the female reproductive environment has lagged behind similar studies of male contributions to the ejaculate (24).

Investigations with *D. melanogaster* have demonstrated essential contributions of the FRT glands and, by extension, glandular secretions to sperm migration into the storage organs, sperm survival in storage, and the regulation of ovulation and oviposition (74, 76, 77). In this first examination of the *Drosophila* FRT fluid proteome, we show that the fluid comprised a subset of the proteins identified in the FRT tissues, which exhibit distinct abundance patterns and postmating responses. Consistent with the few studies of the FRT fluid in other insect species, the *D. melanogaster* fluid proteome was enriched for glycolytic pathways hypothesized to support sperm survival (68, 71) and proteolytic proteins hypothesized to contribute to ejaculate processing and modification (9, 12, 71, 109). The fluid also exhibited pronounced mating-induced compositional changes that were substantively different from those of the tissue proteome. This integration of FRT fluid proteome dynamics with spatiotemporal patterns of gene expression across the FRT tissues (83) provides a foundation for future molecular investigations of the ejaculate–female interactions.

Our ability to compare the proteomic data with patterns of FRT gene expression (83) allowed us to begin to resolve how FRT tissues regulate extracellular luminal fluid composition. Notably, abundant fluid proteins tended to have higher and broader patterns of expression across the FRT. Those proteins may constitute a core fluid proteome to which all tissues contribute (110), which is consistent with the evidence that most FRT tissues have secretory capacity (65, 78, 111) and the identification of FRT genes that are highly expressed across multiple FRT tissues (83). The FRT tissues may contribute to the fluid through a variety of secretory mechanisms, and our results are useful in beginning to identify these pathways. Consistent with the prediction that merocrine secretion occurs in the exocrine glands of the FRT (*i.e.*, the spermatheca and parovaria), we identified an enrichment of vesicle proteins in the fluid. However, we did not observe an enrichment of proteins with secretion signals. One possible explanation for this discrepancy is that the secreted products have greater regional specificity with lower overall abundance, making them recalcitrant to proteomic identification with current techniques. The secreted proteins are also often heavily glycosylated, which could have precluded their identification. Alternative mechanisms, such as apocrine and holocrine secretion, could also account for the observed fluid composition and dynamics. In particular, the enrichments of intracellular cytoplasmic protein contents, such as translational machinery among identified fluid proteins, support the contribution of apocrine or holocrine secretion to the fluid. We note that the support for these alternative secretory pathways has been found in other invertebrate male reproductive systems (26, 58, 61) and that intracellular proteins have been identified in other studies of both insect and mammalian FRT fluid (43, 68, 71). Ultimately, more refined genetic and cell biology approaches will be needed to delineate the respective contributions of these secretory pathways to the fluid composition. In addition, targeted proteomics and immunofluorescence microscopy will be critical for evaluating fluid heterogeneity across FRT microenvironments that may make distinct functional contributions to reproduction (as seen with vesicles, neuromodulators, and immune response proteins; (79, 80, 81)).

Genes with tissue-specific FRT expression contributed substantially to fluid composition and postmating responses. This relationship was especially pronounced for the FRT-associated fat body. We hypothesize that the female reproductive fat body, which is organized in *D. melanogaster* as spatially discrete clusters of cells surrounding the spermatheca and parovaria, possesses dedicated roles in the establishment and dynamics of the FRT fluid. The spatial and functional heterogeneity of abdominal fat body populations is supported by recent single-cell analyses demonstrating that the fat body cells form clusters with stereotypical expression profiles and disparate postmating responses (112). A specific role of the reproductive fat body as a source of nutrients transferred to the FRT lumen in insects has previously been suggested with regard to both sperm storage in reduviid bugs (113) and embryogenesis in the viviparous tsetse fly (114). The unexpectedly large contribution of the fat body to the fluid composition identified here implicates the fat body as a possible mediator of the female–ejaculate interactions in *Drosophila*, in addition to its role in oogenesis (115). Further mechanistic research is required to elucidate the secretory mechanisms responsible for the trafficking of fat body products into the extracellular environment of the FRT.

Changes in the FRT after mating are hypothesized to facilitate ejaculate–female interactions, including those required for sperm storage, SFP and sperm modification, ovulation, and oviposition (reviewed in (110)). Such postmating responses can additionally facilitate sexual selection or contribute to reproductive barriers between divergent populations or species (6, 19, 23). For example, a recent proteomic study found significant differences in the extent of FRT postmating response between two sibling species that exhibit postmating prezygotic reproductive barriers (62, 72). The greater postmating changes observed in *Drosophila mauritiana* relative to *Drosophila simulans* were on par with the *D. melanogaster* FRT tissue response observed here, which suggests that it reflects the ancestral condition in this clade (62). Nonetheless, our present results reveal that the FRT tissue response to mating pales in comparison with that of the FRT fluid.

It is somewhat surprising that the prominent postmating changes in the fluid did not correspond to contemporaneous changes in the FRT tissue proteome. This pattern may reflect the "poised state" of the FRT allowing for a rapid response to mating (110), potentially through increased secretory activity. It is conceivable that secretory activity occurs within a restricted set of cells and can substantively change the composition of fluid without comparable quantitative proteome shifts across the full repertoire of FRT tissues. However, our results also suggest that fluid proteins may be more efficient to translate because of their shorter average length. As such, they may be rapidly replenished to return the FRT tissues to a “poised” state for subsequent mating. Regardless, it seems likely that tissue and fluid proteome dynamics operate at disparate timescales. Finally, we also note that postmating fluid proteome dynamics may also reflect protein processing or degradation, as the fluid is likely to be in direct contact with proteolytic SFPs (1).

Given its central role in mediating ejaculate–female interactions that determine reproductive outcomes, the FRT fluid should be a priority of future investigations. This is especially true in relation to intraspecific and interspecific compositional variation and of how such variation corresponds to reproductive outcomes, including fertility, paternity, and female postmating responses. In particular, expanding on the time points characterized here to establish a timeline of FRT tissue and fluid proteome changes after mating would allow for a more refined interpretation of protein changes corresponding to physiological events of interest. In addition, it is important to similarly characterize the presence and temporal dynamics of the nonprotein composition of the fluid, including ions, metabolites, amino acids, prostaglandins, and hormones.

## Data availability

Raw mass spectrometry data are available *via* the ProteomeXchange Consortium (FRT tissue and fluid: PXD025085 and isotopic labeling: PXD025072). The analysis code is available on GitHub at https://github.com/CEMcDonoughGoldstein/FRT.TissueFluid.Proteome.

## Supplemental data

This article contains supplemental data.

## Conflict of interest

The authors declare no competing interests.

## Supplemental data

Supplemental Table S1 Protein abundance data and differential abundance analysis for FRT tissue and fluid proteome

Supplemental Table S2 Peptide spectral matches (PSMs) for the isotopically labeled whole-fly bodies to check the efficiency of labeling

Supplemental Table S3 GO functional enrichments for the identified proteins

Supplemental Table S4 Functional enrichments for tissue-biased and fluid-biased proteins identified in the protein-interaction networks.

Supplemental Figure S1 **Sample banding patterns from 2D gel of FRT (*A*) tissue and (*B*) fluid.***C*, hierarchical clustering demonstrates high correlations within sample types and time points.

Supplemental Figure S2 **Comparison of protein abundance based on the identification in FRT tissue and fluid.***A*, fluid proteins (*i.e.*, those identified in both the tissue and fluid) were more abundant on average in the fluid proteome. *B*, rank-mean plot based on tissue protein abundance demonstrates that although fluid proteins are found throughout the entire protein abundance range, they tend to be among the most abundant (tissue proteins: *green* and fluid proteins: *purple*). *C*, fluid-only proteins were less abundant than fluid proteins also identified in the FRT tissue samples.

Supplemental Figure S3 **Codon bias of differentially abundant fluid proteins.** Fluid proteins that increased or decreased in abundance were not encoded by genes with significant differences in codon bias, as measured by ENCprime.

Supplemental Figure S4 **Relationship between postmating protein abundance, fluid abundance changes, and FRT tissue gene expression changes in the same time frame (unmated and 6 h after mating).** Only significant correlations (*p* < 0.05) with tissues are shown: the (*A*) bursa, (*B*) oviduct, (*C*) parovaria, and (*D*) fat body. Gene expression changes in the FRT tissues are positively correlated to protein abundance changes, whereas the fat body is negatively correlated.
