# Supplementary figures and images for: Pronounced Postmating Response in the Drosophila Female Reproductive Tract Fluid Proteome
Source: Mol Cell Proteomics. 2021 Sep 29;20:100156. doi: 10.1016/j.mcpro.2021.100156 (PMC9357439; doi:10.1016/j.mcpro.2021.100156)

Fig. S1

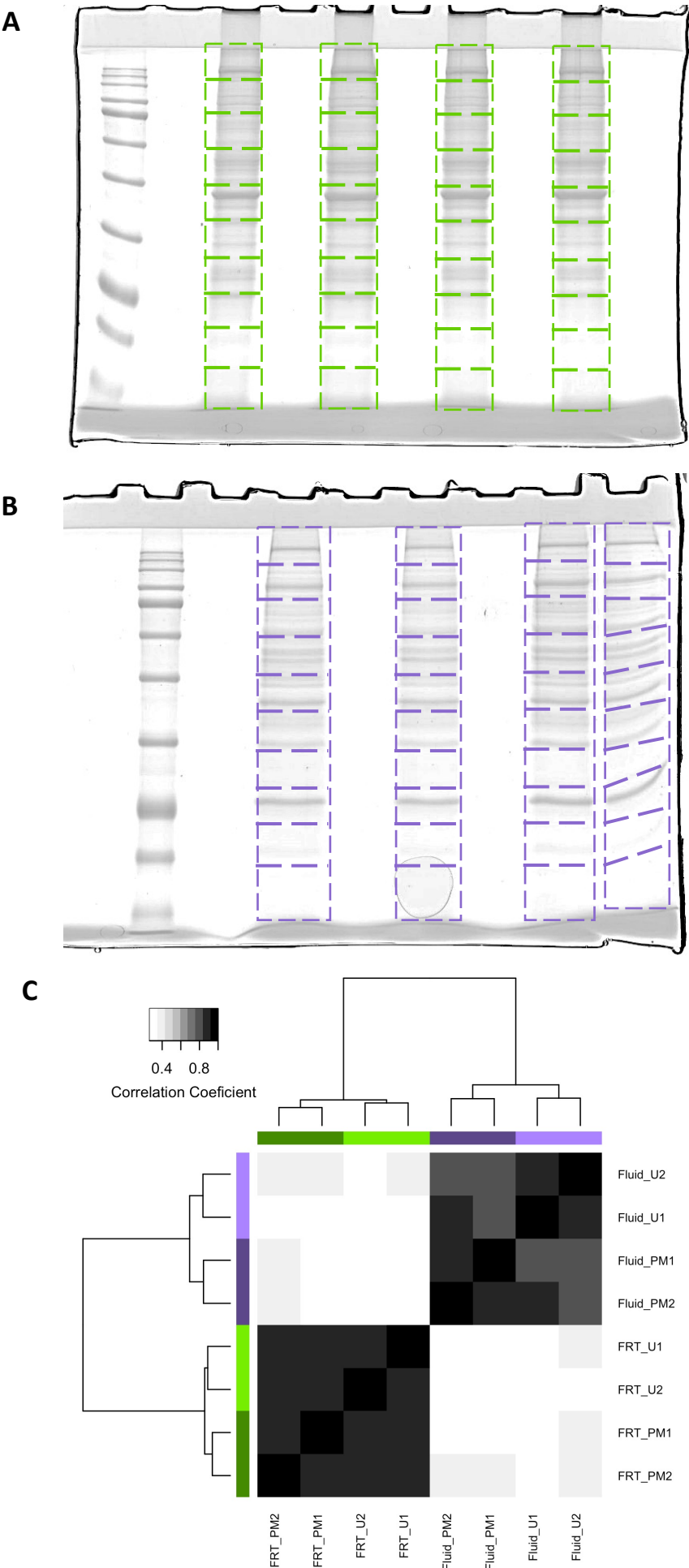

Supplement: Supplemental Figure S1 — Sample banding patterns from 2D gel of FRT (A) tissue and (B) fluid.C, hierarchical clustering demonstrates high correlations within sample types and time points. [file mmc5.pdf]

Fig. S2

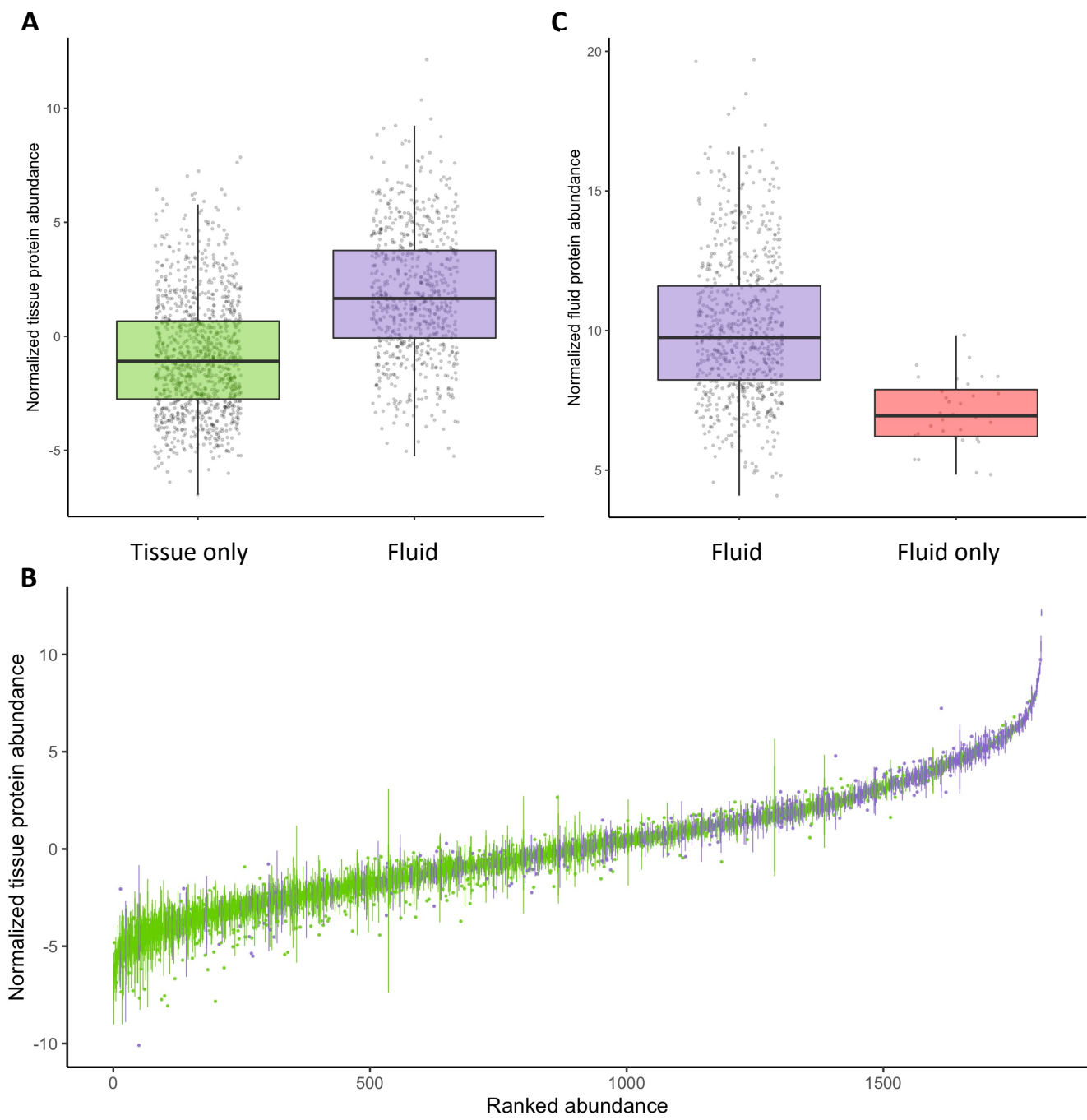

Supplement: Supplemental Figure S2 — Comparison of protein abundance based on the identification in FRT tissue and fluid.A, fluid proteins (i.e., those identified in both the tissue and fluid) were more abundant on average in the fluid proteome. B, rank-mean plot based on tissue protein abundance demonstrates that although fluid proteins are found throughout the entire protein abundance range, they tend to be among the most abundant (tissue proteins: green and fluid proteins: purple). C, fluid-only proteins were less abundant than fluid proteins also identified in the FRT tissue samples. [file mmc6.pdf]

Fig. S3

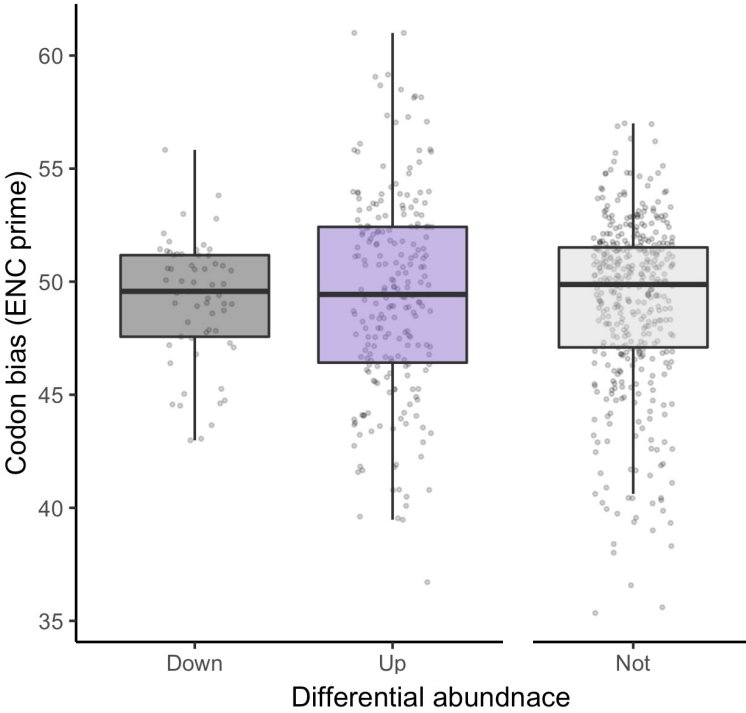

Supplement: Supplemental Figure S3 — Codon bias of differentially abundant fluid proteins. Fluid proteins that increased or decreased in abundance were not encoded by genes with significant differences in codon bias, as measured by ENCprime. [file mmc7.pdf]

Fig. S4

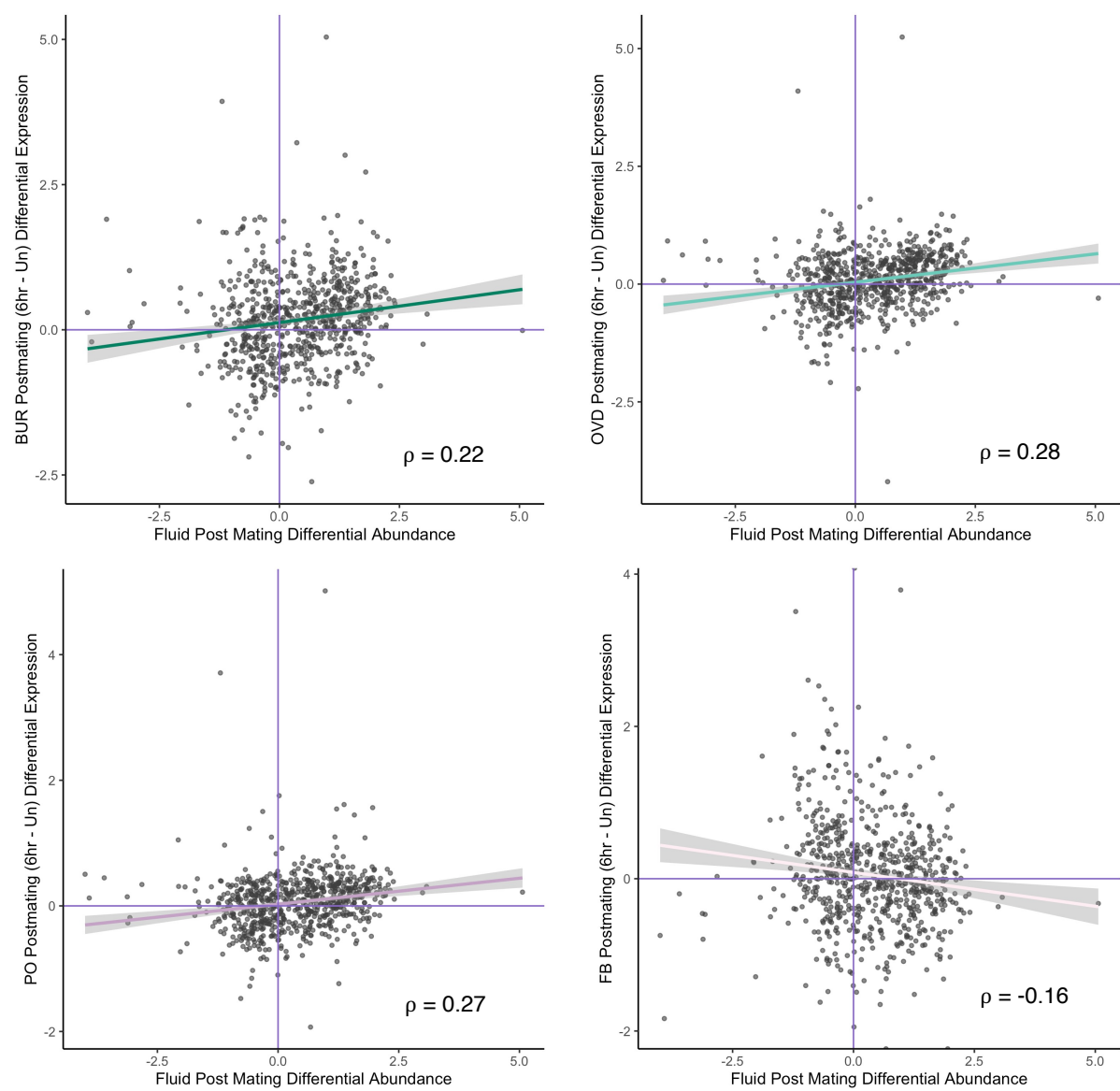

Supplement: Supplemental Figure S4 — Relationship between postmating protein abundance, fluid abundance changes, and FRT tissue gene expression changes in the same time frame (unmated and 6 h after mating). Only significant correlations (p < 0.05) with tissues are shown: the (A) bursa, (B) oviduct, (C) parovaria, and (D) fat body. Gene expression changes in the FRT tissues are positively correlated to protein abundance changes, whereas the fat body is negatively correlated. [file mmc8.pdf]
